# Direction selectivity in the larval zebrafish tectum is mediated by asymmetric inhibition

**DOI:** 10.3389/fncir.2012.00059

**Published:** 2012-09-04

**Authors:** Abhinav Grama, Florian Engert

**Affiliations:** Department of Molecular and Cellular Biology, Harvard UniversityCambridge, MA, USA

**Keywords:** direction selectivity, tectum, vision, asymmetric inhibition, zebrafish

## Abstract

The extraction of the direction of motion is an important computation performed by many sensory systems and in particular, the mechanism by which direction-selective retinal ganglion cells (DS-RGCs) in the retina acquire their selective properties, has been studied extensively. However, whether DS-RGCs simply relay this information to downstream areas or whether additional and potentially *de novo* processing occurs in these recipient structures is a matter of great interest. Neurons in the larval zebrafish tectum, the largest retino-recipent area in this animal, show direction-selective (DS) responses to moving visual stimuli but how these properties are acquired is still unknown. In order to study this, we first used two-photon calcium imaging to classify the population responses of tectal cells to bars moving at different speeds and in different directions. Subsequently, we performed *in vivo* whole cell electrophysiology on these DS tectal neurons and we found that their inhibitory inputs were strongly biased toward the null direction of motion, whereas the excitatory inputs showed little selectivity. In addition, we found that excitatory currents evoked by a stimulus moving in the preferred direction occurred before the inhibitory currents whereas a stimulus moving in the null direction evoked currents in the reverse temporal order. The membrane potential modulations resulting from these currents were enhanced by the spike generation mechanism to generate amplified direction selectivity in the spike output. Thus, our results implicate a local inhibitory circuit in generating direction selectivity in tectal neurons.

## Introduction

The ability to encode the direction of motion of a stimulus is an important feature extraction from the dynamic world we live in. Specialized neurons across many sensory systems serve exactly this purpose (Fried et al., 2002; Priebe and Ferster, 2005; Wilent and Contreras, 2005; Chacron and Fortune, 2010; Wang et al., 2010; Ye et al., 2010). The optic tectum, better known as the *superior colliculus* in mammals, is a multi-layered structure that integrates information from different sensory modalities (Stein et al., 2009; Deeg et al., 2009) and has neurons that are direction-selective (DS) for moving visual stimuli (Rhoades and Chalupa, 1976; Engert et al., 2002; Niell and Smith, 2005; Wang et al., 2010). DS neurons have also been found in larval zebrafish tectum (Niell and Smith, 2005; Ramdya and Engert, 2008), and this property is a likely mediator for the visual goal-directed behaviors that this animal performs.

The mechanism underlying direction selectivity has received a lot of attention with a specific focus on the retina across different invertebrates and vertebrates (Barlow and Levick, 1965; Joesch et al., 2008; Kim et al., 2008; see Borst and Euler, 2011 for review). Rather than one conserved motif, a variety of mechanisms have been reported by studies so far. The best understood DS circuit is that of mouse retinal ganglion cells (RGCs) (Weng et al., 2005; Briggman et al., 2011; Wei et al., 2011). It was shown for On-Off direction-selective RGCs (DS-RGCs) that inhibition is biased toward the null direction of motion and excitation biased toward the preferred. Also, inhibition arrives before the excitation for the null direction, thus preventing spiking, whereas the reverse happens for the preferred (Fried et al., 2002; Weng et al., 2005). This is mediated by a starburst amacrine cell that feeds inhibition to the On-Off DS-RGCs. A different mechanism was reported in the visual and the auditory cortex, where both excitation and inhibition were biased toward the preferred direction, but the latency relationship was similar to that in the retina (Zhang et al., 2003; Priebe and Ferster, 2005). In the auditory inferior colliculus and the barrel cortex, only the latency relationship was shown to be responsible for direction selectivity, with the magnitudes of excitatory and inhibitory inputs being the same for both directions (Wilent and Contreras, 2005; Kuo and Wu, 2012).

In the larval zebrafish tectum, the precise mechanism of direction selectivity still remains unknown. The fact that DS-RGC responses were recorded from axon terminals in a closely related teleost, adult goldfish (Maximov et al., 2005), and that DS-RGCs have also been shown to project to the tectum in other vertebrates (Kim et al., 2008; Huberman et al., 2009) suggests that tectal cells can already receive pre-processed DS input directly from the retina. Thus, the most parsimonious way to explain direction selectivity in tectal neurons, or for that matter in all retino-recipient areas across model systems, would be a direct relay from DS-RGCs to their specific downstream targets. However, in the mammalian visual system in particular there is accumulative evidence that the DS properties of higher order neurons, like those found in the visual cortex, are not explained by a simple feed-forward circuit from DS-RGCs, but are rather the product of local and intra-cortical processing (Priebe and Ferster, 2005; Priebe et al., 2010). Similarly, in the larval zebrafish, a previous study reported that it is possible for the tectum to extract direction selectivity independent of DS-RGCs, with a local blockade of inhibitory transmission causing a drop in selectivity (Ramdya and Engert, 2008). However, the details of how this information is extracted are still unclear.

Here we use a combination of two-photon calcium imaging and *in vivo* whole cell patch-clamping to address this question. We found that many cells showed strong direction selectivity as well as a preference for speed. Surprisingly, we found that the input excitatory currents were only weakly tuned to the direction of motion, whereas, the inhibitory currents were strongly biased toward the null direction. When we examined the latency between the excitatory and inhibitory currents, we found that inhibition tended to precede excitation in the null direction and the reverse was true for the preferred direction. The membrane potential change resulting from the interaction of the excitatory and inhibitory inputs was further amplified by the spike generation mechanism to generate a stronger DS spike output. Thus, our results point toward an inhibitory model of direction selectivity, a motif that seems similar to that found in the retina (Fried et al., 2002; Weng et al., 2005).

## Materials and methods

### Zebrafish rearing conditions

*Nacre* (–/–) zebrafish used in this study were raised at 28°C on a 14 h on/10 h off light cycle in E3 solution (5 mM NaCl, 0.17 mM KCl, 0.33 mM CaCl2, and 0.33 mM MgSO4). All experiments were approved by Harvard University's Standing Committee on the Use of Animals in Research and Training.

### *In vivo* calcium imaging

Calcium imaging experiments were carried out at 6–8 days post fertilization (dpf). For injections, zebrafish were anaesthetized using 0.02% MS222 and mounted in 1.5% low-melting agarose. 1 mM Oregon Green BAPTA-1 AM (Molecular Probes) Ester dissolved in DMSO with 20% pluronic acid (vol/vol) as well as E3 solution (5 mM NaCl, 0.17 mM KCl, 0.33 mM CaCl2 and 0.33 mM MgSO4) containing 100 mM Alexa Fluor 594 (Molecular Probes) was bolus injected into the tectal neuropil of the right tectum with 10–50 ms pulses at 1 psi using a PV820 Pneumatic PicoPump (World Precision Instruments). Fish were freed and allowed to recover for at least 1 h before imaging. For imaging, zebrafish were re-mounted in agarose on a custom-built cylinder shaped acrylic chamber. Calcium imaging experiments were carried out using a custom-built two-photon microscope coupled to a Mai Tai (Spectra-Physics) mode locked Ti:Sapphire laser (950 nm) and a 20× water-immersion objective with a 0.95 numerical aperture (Olympus). Images were acquired at 1 Hz.

### *In vivo* electrophysiology

For recordings, larval zebrafish at 6–8 dpf were paralyzed in alpha-Bungarotoxin (Invitrogen) at 1 mg/ml in zebrafish external solution (NaCl 134 mM; KCl 2.9 mM; CaCl2 2.1 mM; MgCl2 1.2 mM; glucose 10 mM; HEPES 10 mM; pH 7.8, 290 mOsm) with 0.02% MS222 for 10 min. Following this, the anaesthetized and paralyzed fish were mounted on a custom chamber using insect pins. To access the right tectum, the overlying skin was removed using a sharpened tungsten needle. Electrophysiological recordings were performed at room temperature using an Axopatch 200B (Axon Instruments) patch-clamp amplifier. Data were sampled at 5 kHz and filtered at 2 kHz with a National Instruments data acquisition board. Borosilicate glass capillary micropipettes (World Precision Instruments) were pulled for a resistance of 12–15 MΩ and were filled with a K-gluconate internal solution (K-gluconate 115 mM; KCl 15 mM; MgCl2 2 mM; HEPES 10 mM; EGTA 10 mM; Na2ATP 4 mM; pH 7.2, 280 mOsm). Tectal cells in the medio-lateral tectum were patched under infrared illumination. The membrane properties of these tectal cells were estimated in a separate set of experiments (input resistance = 3.2± 1.2 GΩ, tau = 21± 7 msec, *n* = 7 cells). Excitatory post-synaptic currents (EPSCs) were recorded in whole-cell configuration with voltage-clamp at −70 mV. Inhibitory post-synaptic currents (IPSCs) were recorded at a holding potential of 0 mV. The membrane potential was subsequently recorded in current clamp mode with the resting potential set to −50 mV.

### Visual stimulation

Visual stimuli were presented using a DLP projector (Optoma) filtered by a #29 Wratten filter (Kodak). Images were passed through a 0.42X wide-angle lens (Kenko) and bottom-projected onto a screen encompassing 120° of visual angle. High contrast 3° width vertical bars were presented on a dark background in all experiments and moved caudo-rostrally (CR) or rostro-caudally (RC). For calcium imaging, the speed of the bar was varied from 10–100°s in 10° increments and in a pseudo-random fashion, with three repeats for each speed and direction. For electrophysiological experiments, bars moved in the CR and RC directions at 60°/s (five repeats in each direction, pseudo-randomly interleaved).

### Data analysis

Regions of interest (ROIs) over cells were manually chosen from an average image of the movies and the fluorescence signals from these cells were converted to a (delta f)/f (df/f) value. Only neurons that showed calcium activity (spontaneous or stimulus locked) were further analyzed. The peak df/f in a window following the stimulus presentation (stimulus presentation time +5 s) was chosen as the response of a neuron to the stimulus. To test for response to motion, a *t*-test was performed between the peak df/f of the baseline and that of the response periods and only neurons that showed a significant difference (*p* < 0.005) were considered motion-responsive. The responses of neurons to different speeds were assessed with ANOVA and only cells with *p* < 0.05 were considered as modulated by speed. The responses to different speeds were fit with a smoothing spline and the maximum of this fit gave the preferred speed. Direction selectivity was quantified by a ratiometric direction selectivity index (DSI), where Resp_CR_ indicates response (either df/f or electrophysiological) in the CR direction, similarly for others.

$$\text{DSI}=\frac{\left({\text{Resp}}_{\text{CR}}-{\text{Resp}}_{\text{RC}}\right)}{\left({\text{Resp}}_{\text{CR}}+{\text{Resp}}_{\text{RC}}\right)}$$

The responses in voltage clamp experiments were calculated as the integrated charge of the mean neuronal discharge in response to the stimulus. For current clamp experiments, the spikes were first counted and then filtered out to give the membrane potential modulation. The membrane potential response was taken to be the maximum of the baseline subtracted mean trace in the response period. To calculate the latency between the excitatory and inhibitory currents, we fit the smoothed average currents with a double exponential function (Naumann et al., 2010) and computed the latency as the half maximal point of the fit.

## Results

### Two-photon calcium imaging of responses to moving bars

**Figure 1:**
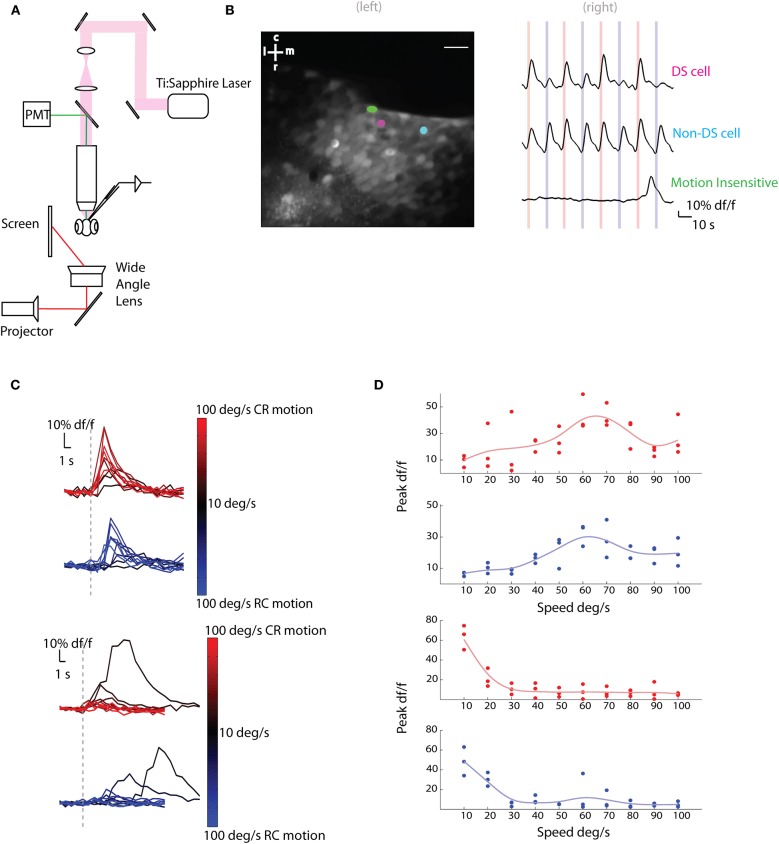
**Two-photon calcium imaging of tectal responses to moving bars. (A)** Schematic of the experimental setup. Larval zebrafish were placed in a custom chamber (chamber not shown) and presented bars moving caudo-rostrally (CR) or rostro-caudally (RC) to the left eye. Calcium imaging and electrophysiological recordings were performed on the right tectum. **(B)** (Left) Average image of a two-photon stack with three neurons highlighted as ROIs. Scale bar represents 20 μm (r, rostral; c, caudal; m, medial; l, lateral; 85% of active cells in this example fish were sensitive to motion). (Right) Delta f/f (df/f) traces of the highlighted neurons. Red lines indicate stimulus moving in the CR direction and blue lines indicate RC direction at 60°/s. The cell in magenta was direction selective (DS) for the CR direction, the cell in cyan was non-DS and the cell in green was insensitive to motion. **(C,D)** In **(C)**, Average df/f responses (*n* = 3 trials) of two cells to different speeds are shown. Red indicates motion in the CR direction and blue motion in the RC direction. Darker colors imply slower speeds and lighter colors faster speeds. The responses to slowly moving bars are delayed with respect to the onset of the stimulus as the time taken for the bar to enter the receptive fields of the neurons is longer. This is seen in the second cell's (bottom) response to slower stimuli. In **(D)**, the single trial peak df/f responses (filled circles) of the same two cells to bars moving in the CR (red) or RC (blue) directions at different speeds are shown. The responses were fit with a smoothing spline (light blue or red lines) and the preferred speed was estimated. The cell on top shows band-pass tuning, while the cell on the bottom shows low pass tuning.

We first characterized the responses of tectal cells to moving bars by loading these cells with a synthetic, membrane permeable calcium indicator, Oregon green bapta-1-AM (OGB1-AM). This allowed us to record calcium signals from hundreds of neurons simultaneously, which have been shown to be a reasonable proxy for measuring action potentials in this preparation (Niell and Smith, 2005). To immobilize fish for imaging purposes, animals were restrained in agarose and visual stimuli were presented—to one eye—while the contralateral tectum was imaged on a custom two-photon microscope (Figure 1A).

**Figure 2:**
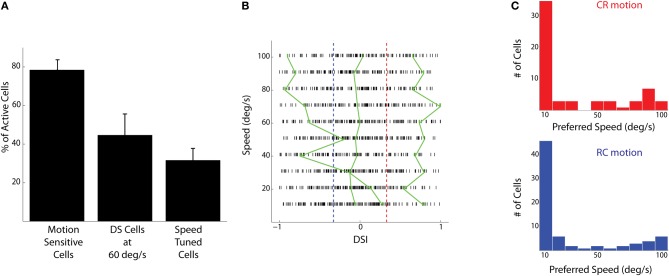
**Population responses to bars moving at different speeds and directions. (A)** The percentage of active cells that are motion sensitive, that are direction selective at a speed of 60°/s, and that show responses modulated by speed (mean ± SD). **(B)** Direction selectivity indices (DSIs) of individual tectal cells at different speeds are shown as raster ticks. Cells to the right of the red dashed line are CR selective and cells to the left of the blue line are RC selective. The green lines show the dependence of DSI on the speed of the stimulus for three cells. The cells shown have low (SD = 0.04), medium (SD = 0.12), and high (SD = 0.44) variability of DSIs at different stimulus speeds. **(C)** The histogram of preferred speeds for all speed tuned cells in the CR direction (red, *n* = 61 cells) and RC direction (blue, *n* = 73 cells).

Stimuli consisted of 3° vertical bars moving CR or RC at different speeds (10–100°/s). Responses of motion sensitive cells (Figure 1B) were analyzed for the different speeds and directions presented. A fraction of the active cells (31±6% of *n* = 327 cells from five fish) were found to be modulated by the speed of the moving bars (Figures 1C,D, 2A). To find the preferred speeds of these cells, the responses were fit with a smoothing spline and the speed that gave the maximum response was estimated (Figure 1D). There seemed to be a preference for low speeds for both the CR and RC directions of motion (Figure 2C).

To quantify the responses of the tectal cells to either direction, a (DSI) was calculated based on the peak calcium responses. With this formulation, highly CR selective neurons would have a DSI of +1, highly RC selective neurons would have a DSI of −1 and non-selective neurons would have a DSI of 0. The DSIs of tectal cells spanned the entire range from −1 to 1 for all the speeds presented (Figure 2B). At 60°/s (the speed used for electrophysiological recordings), about 44 ± 10% of the active cells, or more than half the number of motion sensitive cells, were found to have a |DSI| > 0.33 (Figure 2A). This implies that a cell is twice as responsive to motion in one direction than the other. This finding is in agreement with that of a previous study, which measured responses of tectal cells to moving spots (Niell and Smith, 2005).

### Direction selectivity of excitation and inhibition

**Figure 3:**
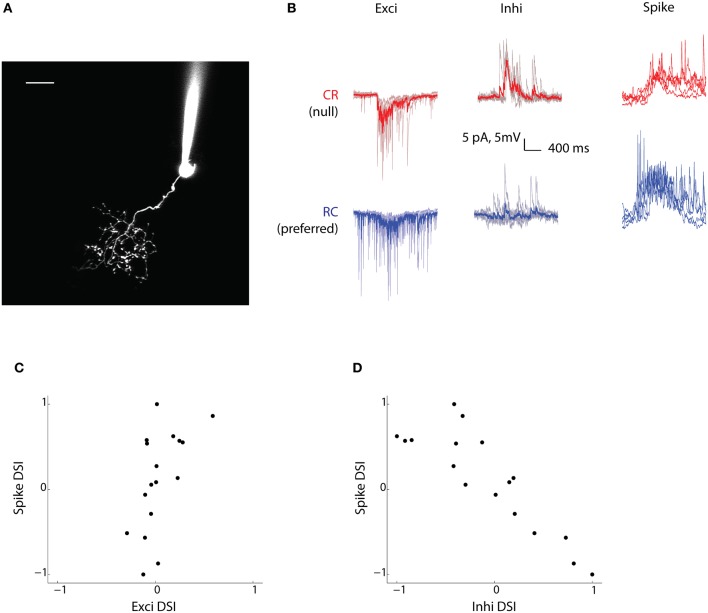
**Inhibitory currents are biased toward the null direction of motion. (A)** Two-photon fluorescence image of a tectal neuron filled with dye (Alexa 594) from the recording electrode. Tectal neurons project dendritic arbors into a neuropil where they receive retinal ganglion cell axonal input from the contralateral eye. Scale bar represents 10 μm. **(B)** Voltage clamp (Exci and Inhi) and current clamp (Spike) recordings of a tectal cell's response (for the duration the stimulus was presented) to bars moving in the CR direction (red) and the RC direction (blue). For the currents, the mean trace (thicker red/blue line) is shown superimposed over five trials (lighter red/blue lines). This cell is RC selective and has strong inhibition from the null CR direction. **(C** and **D)** Spike-DSI for all cells (*n* = 17) plotted vs Exci-DSI and Inhi-DSI, respectively.

With quite a large fraction of tectal cells showing direction selectivity, one of the more pertinent questions that follow concerns the mechanism that leads to this property being established. To address this, we performed *in vivo* whole cell patch-clamp recordings of cells in the *stratum periventriculare* (SPV) of the tectum, the region where over 95% of all tectal cells reside (Niell and Smith, 2005) (Figure 3A). SPV cells are unipolar in morphology; they send dendrites into the tectal neuropil where they synapse with RGC axons (Scott and Baier, 2009). SPV cells in the right tectum were patched under infrared illumination while vertical bars moving in either the CR or RC direction were presented to the left eye. Given the limited recording time for electrophysiology, we didn't measure the preferred speed of the neurons and used a fixed speed (60°/s) while probing DS responses for all cells. There is a possibility that the profile of the synaptic currents might vary at different speeds for individual neurons and ideally one would like to characterize DSI tuning for all possible speeds for each neuron. However, the population data from calcium imaging suggests that there isn't a strong dependence of DSI values on speed (the average standard deviation of DSIs for all cells with responses to at least three speeds was 0.2) and therefore these effects are probably not substantial. We isolated the excitatory and inhibitory currents by clamping the cells at −70 mV and 0 mV, respectively (Smear et al., 2007; Zhang et al., 2011). Subsequently, we switched to current clamp mode and recorded the spiking output of these cells. In response to the moving bars, the tectal cells showed excitatory and inhibitory currents that were time locked to the spikes and membrane potential changes (Figure 3B). We recorded from 17 neurons (from 15 fish) whose responses spanned the range from highly CR selective to highly RC selective.

To look at the contribution of excitatory and inhibitory currents to the spiking output of the cells in response to moving bars, excitatory (Exci), inhibitory (Inhi), and Spike DSIs were calculated. When we compared the Exci-DSI with the Spike-DSI (Figure 3C), we were surprised to find that the excitatory currents were not well tuned to the direction of motion, despite showing a significant correlation (*R*^2^= 0.32, *p* = 0.02, *n* = 17). For most of the cells the Exci-DSI did not exceed 0.33. Thus, our data suggests that excitatory currents don't play a major role in generating direction selectivity in most tectal cells.

In contrast, when the relationship between the inhibitory currents and the spike output was examined, a strong anti-correlation was found (Figure 3D, *R*^2^ = 0.76, *p* < 0.00001, *n* = 17). This means that for cells with a Spike-DSI close to 1, i.e., CR selective, the Inhi-DSI was close to −1, i.e., the inhibitory currents were heavily biased in the null RC direction. This finding establishes a mechanism for observations made in a previous study (Ramdya and Engert, 2008), where abolishing inhibitory transmission through GABA_A_ receptors resulted in a great reduction in direction selectivity. This arrangement of inhibition coming from the null direction of motion is also seen in the retina, where DS-RGCs receive inhibition in the null direction from starburst amacrine cells (Fried et al., 2002; Weng et al., 2005). Our results strongly implicate a local tectal computation of direction selectivity via inhibition.

### Timing of excitation and inhibition

**Figure 4:**
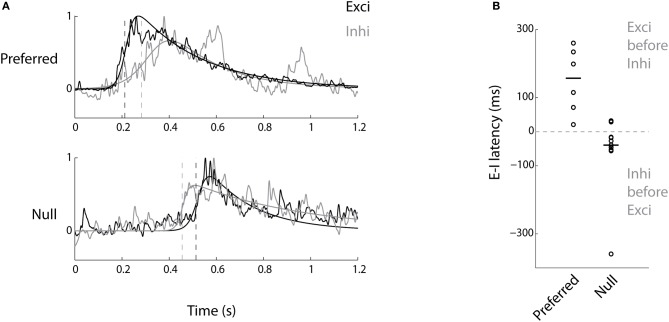
**Inhibition precedes excitation in the null direction and follows it in the preferred direction. (A)** Average excitatory (black) and inhibitory (gray) current profiles from a DS cell to bars in the preferred and the null directions are shown after normalization to illustrate the temporal relationship between them. **(B)** The latency between excitatory and inhibitory currents for preferred and null directions for all the DS cells (*n* = 6 preferred, *n* = 11 null, see text). Negative values mean inhibition precedes excitation.

In DS-RGCs, apart from the magnitude of inhibition being biased toward the null direction, there is also an asymmetry in the timing of the inhibitory currents compared to the excitatory currents (Fried et al., 2002; Weng et al., 2005). To test for any such relationship for DS tectal cells, the temporal order of the excitatory and inhibitory currents (|DSI| > 0.33, *n* = 11 cells) was examined. In the null direction, the inhibitory currents preceded the excitatory currents (Figure 4, *p* < 0.05, Wilcoxon's signed rank test, median value = 39 ms, *n* = 11 cells). Thus, the bias in the magnitude of tectal inhibition and its temporal relationship to excitation ensured that there was very little spiking in the null direction. In the preferred direction, the reverse relationship was observed, where the excitatory currents were leading the inhibitory currents, thereby causing the cells to spike (Figure 4, *p* < 0.05, Wilcoxon's signed rank test, median value = 157 ms, *n* = 6 cells). The difference in the onset time of excitatory currents between the preferred and null direction is due to the bar entering the receptive field of the neuron at different times after the stimulus onset, i.e., the time when the bar first appears on the screen. The same reasoning applies to the inhibitory currents. The number of cells examined for latency in the preferred direction (*n* = 6 cells) was less than that for the null direction (*n* = 11 cells) since in quite a few cells there wasn't any appreciable inhibition in the preferred direction (Figure 3B).

### Direction selectivity of membrane potential

**Figure 5:**
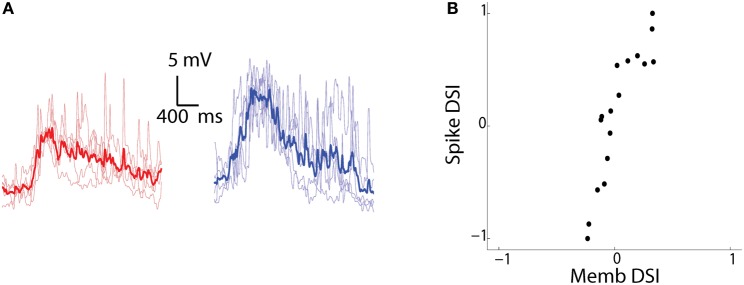
**Sharpening of membrane potential DS by the spike generation mechanism. (A)** Membrane potential recordings in current clamp mode of the same cell as Figure 3B to bars moving in CR (red) and RC (blue) directions. The spikes were filtered out by using a median filter. The mean trace (dark) is superimposed over traces from *n* = 5 individual trials (light). **(B)** Spike-DSI plotted vs Memb-DSI for all the recorded cells (*n* = 17).

To examine the modulation of membrane potential in response to the CR and RC moving bars, we filtered out the spikes (Figure 5A) and calculated a membrane (Memb) DSI. When we compared the Memb-DSI with the Spike-DSI, we found almost a linear relationship (Figure 5B, *R*^2^ = 0.80 *p* < 0.00001, *n* = 17 cells) between the two. There seemed to be an enhancement of the direction selectivity in the spikes when compared to the membrane potential. This phenomenon is also seen in the visual and auditory cortex (Priebe and Ferster, 2005; Ye et al., 2010). The amplification can be attributed to a non-linear effect of the spike threshold (Priebe and Ferster, 2008), which enhances small differences of membrane potential over the threshold to large differences in spike output. Thus, direction selectivity undergoes amplification in tectal cells through the spike generation mechanism.

## Discussion

Our experiments in characterizing the population responses of larval zebrafish tectum confirmed earlier findings about the proportion of DS neurons (Niell and Smith, 2005). Tectal cells showed DSIs spanning the range from −1 to 1 at all speeds presented. In addition, some cells showed responses that were modulated by the speed of the moving bar. Speed tuning has been reported in the mammalian superior colliculus and other visual areas across species such as the area MT and V1 (Rhoades and Chalupa, 1976; Maunsell and Van Essen, 1983; Razak and Pallas, 2005; Priebe et al., 2006), with Razak and Pallas showing an inhibition mediated mechanism for speed tuning in the hamster superior colliculus. Speed encoding cells could be useful feature extractors for moving visual stimuli. In fact, the range of preferred speeds seen in this study matches the speeds at which the image of a paramecium moves on the retina of a hunting zebrafish larva, when it is located at the characteristic striking distance of 1.5 mm away from the fish (Bonini et al., 1986; Bianco et al., 2011). Thus speed encoding cells could aid in goal-directed behaviors such as prey-capture (Ewert et al., 2001; Gahtan et al., 2005).

Another critical feature that needs to be extracted from moving objects is their direction. This is performed across different sensory modalities (Fried et al., 2002; Wilent and Contreras, 2005; Chacron and Fortune, 2010; Ye et al., 2010) and, in the case of vision, even at different stages of the same modality (Fried et al., 2002; Priebe and Ferster, 2005). Models to explain how direction selectivity arises have been in existence for over half a century now (Barlow and Levick, 1965; Reichardt, 1987). Rather than one conserved motif acting across brain areas, there are now many different mechanisms that have been reported. Ranging from biases in excitation and inhibition to just temporal asymmetries (Fried et al., 2002; Zhang et al., 2003; Priebe and Ferster, 2005; Kuo and Wu, 2012), these mechanisms have served to demonstrate the range of evolutionary answers to a fundamental computational problem of sensory processing.

**Figure 6:**
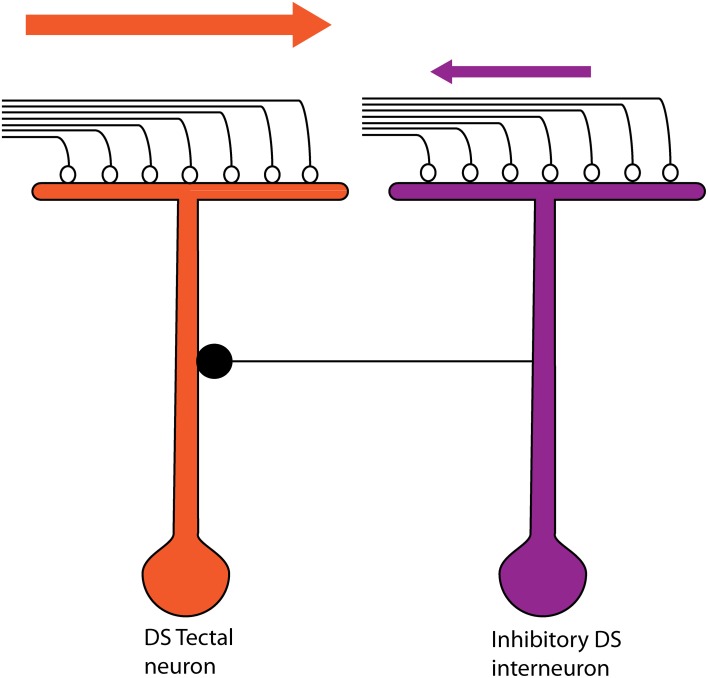
**The proposed model for the direction-selective circuit in the larval zebrafish tectum.** The DS tectal neuron receives excitatory inputs from RGCs (open circles). This neuron also receives an inhibitory input (filled circle) from an interneuron that is topographically shifted toward the null direction of the DS tectal neuron. The interneuron is itself direction selective but for the opposite direction to that of the DS tectal neuron being considered. Thus, if a bar were to move in the preferred direction of the DS tectal neuron (orange arrow), the excitatory currents from the RGC terminals would arrive before the input from the Inhibitory DS interneuron. Also, the inhibition from the interneuron would be low. In the null direction for the DS tectal neuron, higher inhibition from the Inhibitory DS interneuron would arrive before the excitatory inputs from the RGC terminals.

Tectal cells show robust DS responses but the mechanism underlying this computation is so far not completely understood. Ablation studies in the mammalian superior colliculus pointed to a role of cortico-tectal connectivity in this process (Wickelgren and Sterling, 1969; Rosenquist and Palmer, 1971), but this is unlikely in larval zebrafish as no structure equivalent to the visual cortex has been reported here. DS-RGCs projecting to the tectum (Maximov et al., 2005) appeared to be the most likely source for direction selectivity. To address their contribution, we performed *in vivo* whole cell patch experiments in tectal neurons and examined their input excitatory currents, a majority of which have been shown to be retinal in origin (Zhang et al., 2011). To our surprise, there was only a weak correlation of the direction selectivity of the spiking output with that of the excitatory currents, with only a few cells showing a strong bias of excitatory currents toward the preferred direction. This calls to question the role DS-RGCs play in this circuit. When we examined the inhibitory currents, we found that they were biased toward the null direction of motion. The timing differences between the excitatory and inhibitory currents revealed another level of detail in the DS circuit. We found that the inhibition tended to precede excitation in the null direction whereas the reverse was true for the preferred direction. Thus asymmetries in the magnitude and timing of inhibition seem to underlie the generation of direction selectivity in the larval zebrafish tectum. This is most likely mediated by a DS tectal inhibitory interneuron that is topographically shifted toward the null side (Figure 6). This arrangement ensures that greater inhibition arrives before excitation in the null direction and lesser inhibition after excitation in the preferred direction. Although the individual elements are different, the logic behind this circuit is similar to that seen in the vertebrate retina (Fried et al., 2002; Weng et al., 2005). Thus there seems to be a conserved motif operating in the retina and the zebrafish tectum.

Our results raise interesting questions for future studies on this circuit. The first question would be that of the seeming redundancy of the DS computation in a part of the brain that already receives a pre-computed input. Given the architecture of the tectum, we would like to argue that the presence of a local computation can be useful. The tectum receives inputs from different sensory modalities that are in topographic register with each other (Deeg et al., 2009). This feature enables a coherent map of the world surrounding the fish to be represented internally. Thus the detection of motion in one sensory dimension could be corroborated with that in the other sensory dimensions owing to such local computations. The second question concerns the presence of DS tectal inhibitory neurons. How do these inhibitory neurons achieve their direction selectivity? Could they be the targets of the DS-RGCs? This study was agnostic about the neurotransmitter phenotype of the recorded cells but this could be addressed in future studies which would lead to further dissection of the DS circuit in the zebrafish tectum. The third question concerns the projection of DS-RGC inputs in the tectum. In fish where the RGC axons from the ipsilateral eye, which normally project to the contralateral tectum, were made to project to the ipsilateral tectum, it was found that DS responses could be elicited by flashing spots of light in sequence to one eye and then the other (Ramdya and Engert, 2008). This experiment showed that direction selectivity in some SPV cells did not need the input from DS-RGCs. Our results show that the input from the retina is only weakly correlated with the DS property of the tectal cells. In the visual cortex, a pioneering study showed that neurons with highly tuned outputs sampled from a broad range of tuned inputs (Jia et al., 2010), the vector sum of which wasn't highly tuned. If the sampling of retinal inputs by tectal cells in anyway resembles the sampling in the visual cortex, then this would account for the weak preference of direction we see in the excitatory currents.

### Conflict of interest statement

The authors declare that the research was conducted in the absence of any commercial or financial relationships that could be construed as a potential conflict of interest.
